# Gait Biomechanics, Symmetry Restoration, and Functional Recovery Following Total Knee Arthroplasty: A Prospective Longitudinal Cohort Study

**DOI:** 10.3390/life16071208

**Published:** 2026-07-21

**Authors:** Giada Baroni, Elena Lucchesini, Roland Fazakas, Dan Fruja, Laura Ioana Bondar, Ana-Liana Bouroș-Tataru, Csongor Toth, Nicoleta Anamaria Pascalau, Liliana-Oana Pobirci, Alexandru Pop

**Affiliations:** 1 Doctoral School of Medicine, “Vasile Goldiș” Western University of Arad, 310025 Arad, Romania; baroni.giada@student.uvvg.ro (G.B.); lucchesini.elena@student.uvvg.ro (E.L.); pop.alexandru@uvvg.ro (A.P.); 2 Department of General Medicine, Faculty of Medicine, “Vasile Goldiș” Western University of Arad, 310025 Arad, Romania; fruja.dan@uvvg.ro; 3 Department of Biology and Life Sciences, Faculty of Medicine, “Vasile Goldiș” Western University of Arad, 310025 Arad, Romania; bondar.laura@uvvg.ro; 4 Doctoral School of Biomedical Sciences, University of Oradea, 410087 Oradea, Romania; csongor.toth@uav.ro; 5 Faculty of Physical Education and Sport, “Aurel Vlaicu” University of Arad, 310130 Arad, Romania; 6 Department of Psycho Neuroscience and Recovery, Faculty of Medicine and Pharmacy, University of Oradea, 410087 Oradea, Romania; nicoleta.pascalau@didactic.uoradea.ro (N.A.P.); opobirci@uoradea.ro (L.-O.P.)

**Keywords:** biomechanics, functional recovery, gait analysis, gait symmetry, knee osteoarthritis, rehabilitation, spatiotemporal parameters, total knee arthroplasty, walking speed, WOMAC

## Abstract

**Background/Objectives:** Total knee arthroplasty (TKA) is the standard surgical treatment for end-stage knee osteoarthritis (KOA); however, restoration of normal gait biomechanics remains an important challenge. Objective assessment of gait recovery may provide valuable information beyond conventional clinical outcome measures. This study aimed to evaluate changes in spatiotemporal gait parameters, symmetry restoration, and functional recovery during the first year following primary TKA, and to identify predictors of postoperative gait performance. **Methods:** A prospective longitudinal cohort study was conducted in 150 patients undergoing primary unilateral TKA for end-stage KOA. Spatiotemporal gait parameters, symmetry-related variables, and functional outcomes were assessed preoperatively and at 3, 6, and 12 months postoperatively. Gait analysis included walking speed, step length, cadence, stance phase symmetry, symmetry index (SI), loading asymmetry, and single-limb support time. Functional evaluation included the Timed Up and Go (TUG) test, the Western Ontario and McMaster Universities Osteoarthritis Index (WOMAC), and the Knee Society Score (KSS). Repeated-measures analysis of variance (ANOVA), Pearson correlation analysis, and multivariate linear regression were performed. **Results:** Significant improvements were observed in all gait and functional parameters throughout follow-up (all *p* < 0.001). Walking speed increased from 0.84 ± 0.16 m/s preoperatively to 1.21 ± 0.18 m/s at 12 months, while step length increased from 49.8 ± 7.4 cm to 64.0 ± 6.4 cm and cadence from 91 ± 10 to 108 ± 8 steps/min. SI improved from 73 ± 11% to 93 ± 6%, whereas loading asymmetry decreased from 18.2 ± 6.1% to 4.8 ± 2.3%. Walking speed demonstrated strong correlations with WOMAC score (r = −0.74, *p* < 0.001) and TUG performance (r = −0.79, *p* < 0.001). Multivariate regression analysis identified preoperative walking speed (β = 0.47, *p* < 0.001) as the strongest predictor of postoperative gait recovery, while age and body mass index (BMI) were significant negative predictors. **Conclusions:** Patients undergoing primary TKA demonstrated substantial improvements in gait biomechanics, symmetry restoration, and functional performance during the first postoperative year. Objective gait assessment was strongly associated with clinical outcomes and may facilitate identification of patients at risk of delayed recovery. Incorporating quantitative gait analysis into postoperative evaluation may support individualized rehabilitation strategies aimed at optimizing functional outcomes following TKA.

## 1. Introduction

Knee osteoarthritis (KOA) is one of the most common degenerative joint disorders worldwide and represents a major cause of pain, disability, and reduced quality of life among older adults [1,2,3,4,5]. Progressive cartilage degeneration, osteophyte formation, and joint inflammation contribute to pain, stiffness, muscle weakness, and impaired mobility, ultimately limiting daily activities and functional independence [6,7,8,9]. With increasing life expectancy and the growing prevalence of obesity, the global burden of KOA continues to rise, creating substantial healthcare and socioeconomic challenges [10,11,12,13,14].

Total knee arthroplasty (TKA) is considered the gold-standard treatment for patients with end-stage KOA who fail to respond to conservative management [15,16,17,18,19]. The procedure has demonstrated excellent outcomes regarding pain relief, correction of deformity, restoration of joint function, and improvement in health-related quality of life [20,21,22]. Consequently, TKA has become one of the most frequently performed orthopedic procedures worldwide, with utilization rates expected to increase further over the coming decades [23,24,25,26,27].

Although pain reduction and functional improvement are well-established benefits of TKA, restoration of normal gait remains an important challenge. Patients with advanced KOA often develop compensatory movement patterns characterized by reduced walking speed, shorter step length, altered cadence, asymmetrical weight-bearing, and impaired lower-limb biomechanics [28,29,30,31,32,33]. While surgery successfully addresses structural joint pathology, complete normalization of gait mechanics may not occur in all patients, and residual abnormalities can persist despite favorable clinical outcomes [34,35,36].

Objective gait analysis has emerged as a valuable tool for evaluating postoperative recovery following TKA. Spatiotemporal gait parameters such as walking speed, step length, and cadence provide quantitative information regarding locomotor function and have been shown to correlate with patient mobility and overall functional performance [37,38,39,40]. Among these parameters, gait symmetry has received increasing attention because it reflects balanced limb loading, coordinated movement patterns, and restoration of biomechanical function. Persistent asymmetry may contribute to inefficient gait, increased energy expenditure, reduced mobility, and excessive loading of the contralateral limb, potentially influencing long-term outcomes [41,42].

Previous studies have reported significant postoperative improvements in gait biomechanics and functional performance following TKA [32,43,44,45,46]. However, the magnitude and timing of recovery remain heterogeneous across investigations. Furthermore, many studies have focused primarily on patient-reported outcomes or isolated biomechanical variables, while relatively few have simultaneously evaluated spatiotemporal gait parameters, symmetry restoration, and functional recovery within a prospective longitudinal framework. In addition, limited evidence is available regarding the clinical and biomechanical factors that predict successful gait recovery after TKA [29,47,48].

A better understanding of postoperative gait restoration is clinically relevant because objective biomechanical measures may identify patients at risk of delayed recovery, facilitate individualized rehabilitation strategies, and improve long-term functional outcomes. Therefore, comprehensive assessment integrating gait biomechanics, symmetry-related parameters, functional outcome measures, and predictive modeling may provide valuable insights into recovery trajectories following TKA.

The present prospective longitudinal cohort study was designed to evaluate changes in gait biomechanics, symmetry restoration, and functional recovery during the first year following primary TKA. The primary objective was to assess longitudinal changes in spatiotemporal gait parameters and symmetry-related outcomes. Secondary objectives were to evaluate changes in functional outcome measures and to investigate the associations between biomechanical and clinical outcomes. An exploratory objective was to identify independent predictors of postoperative gait recovery using multivariate regression analysis.

We hypothesized that: (1) significant improvements would occur in spatiotemporal gait parameters throughout the first postoperative year; (2) gait symmetry and weight-bearing distribution would progressively normalize following surgery; (3) improvements in gait performance would be associated with better functional outcomes; and (4) baseline clinical and biomechanical characteristics would independently predict walking performance at 12 months after TKA.

## 2. Materials and Methods

### 2.1. Study Design and Participants

This prospective longitudinal cohort study was conducted to evaluate changes in gait biomechanics, symmetry restoration, and functional recovery following primary TKA in patients with end-stage KOA. The study was carried out within the Clinical Department of Orthopaedics and Traumatology of the Arad County Emergency Clinical Hospital, Arad, Romania.

The study was conducted between June 2024 and June 2026. This period comprised patient recruitment, prospective follow-up, completion of the scheduled 12-month assessments, data verification, and statistical analysis. Patient recruitment was completed before the end of the study period, allowing all enrolled participants to complete the planned 12-month follow-up before the final analysis.

Participants were eligible for inclusion if they met the following criteria:

- Age ≥ 50 years;
- Diagnosis of advanced primary KOA;
- Kellgren–Lawrence grade III or IV confirmed by radiographic evaluation;
- Scheduled for primary unilateral TKA;
- Ability to ambulate independently before surgery;
- Willingness to participate and comply with the follow-up protocol.

Patients were excluded if they met any of the following criteria:

- Previous lower-limb joint replacement surgery;
- Revision TKA;
- Inflammatory arthropathies (e.g., rheumatoid arthritis);
- Neuromuscular disorders affecting gait;
- Severe cardiovascular disease limiting mobility;
- Active infection at the time of surgery;
- Inability to complete postoperative follow-up assessments.

In addition, the contralateral (non-operated) knee was clinically evaluated during the preoperative assessment. Patients presenting with symptomatic end-stage osteoarthritis requiring bilateral TKA or other musculoskeletal conditions expected to substantially affect gait were not included in the study. The contralateral knee was considered clinically suitable as the reference limb for symmetry analyses. Although mild to moderate degenerative changes could be present, these were not associated with symptoms requiring surgical intervention and were managed conservatively.

A total of 185 patients were screened for eligibility. Following the application of the inclusion and exclusion criteria, 150 patients were enrolled in the study. Baseline demographic and clinical characteristics, including age, sex, body mass index (BMI), affected limb, and radiographic disease severity, were recorded before surgery.

Participants were evaluated preoperatively and subsequently at 3, 6, and 12 months after surgery. During follow-up, three patients were lost before the 6-month assessment and an additional five patients were lost before the 12-month assessment. Consequently, complete 12-month follow-up data were available for 142 patients, corresponding to a follow-up rate of 94.7%.

**Figure 1:**
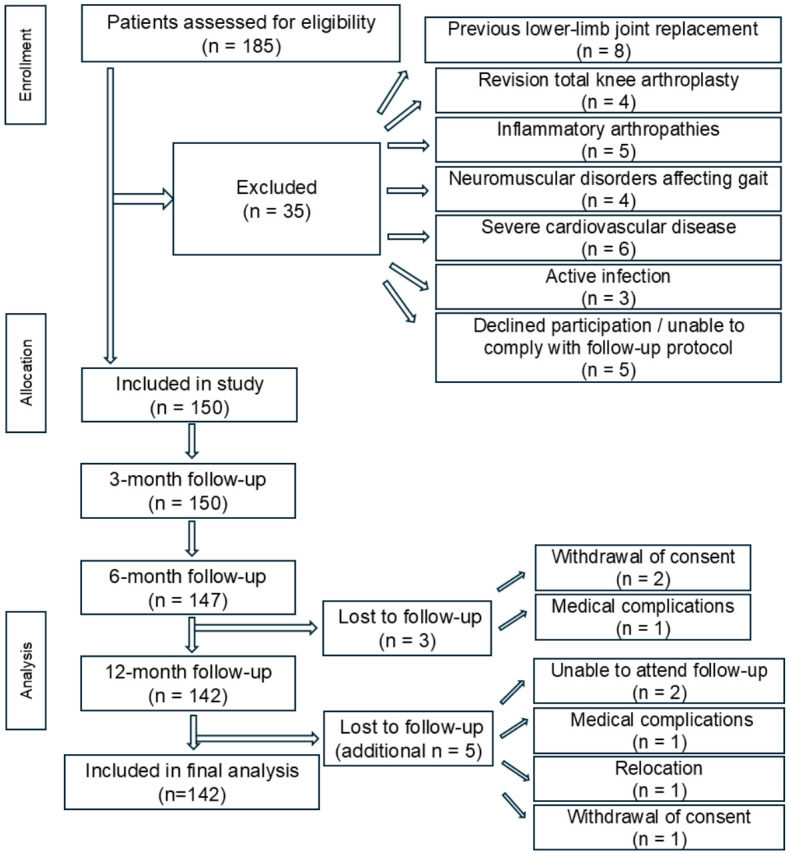
Flow diagram of patient enrollment, follow-up, and inclusion in the final analysis.

All participants underwent a standardized evaluation protocol consisting of spatiotemporal gait analysis, symmetry assessment, and functional outcome measurements at each assessment time point. The patient screening process, follow-up assessments, and inclusion in the final analysis are summarized in Figure 1.

### 2.2. Surgical Procedure

All patients underwent primary unilateral TKA performed by experienced orthopedic surgeons from the Clinical Department of Orthopaedics and Traumatology. Surgical procedures were carried out under spinal or general anesthesia according to individual patient characteristics and anesthesiologist recommendations.

A standard medial parapatellar approach was used in all cases. Following exposure of the knee joint, femoral and tibial bone resections were performed using conventional alignment guides according to the manufacturer’s recommendations. Soft-tissue balancing was carried out to achieve adequate ligament tension and optimal coronal alignment throughout the range of motion.

A cemented posterior-stabilized total knee prosthesis (Persona^®^, Zimmer Biomet, Warsaw, IN, USA) was implanted in all patients. Patellar resurfacing was performed selectively based on intraoperative assessment of the patellofemoral joint. The decision was guided by the presence of advanced patellar cartilage degeneration, severe patellofemoral osteoarthritis, substantial cartilage loss, or irregular patellar articular surfaces, together with the surgeon’s clinical judgment. Patients without these intraoperative findings generally did not undergo patellar resurfacing. After implantation, knee stability, range of motion, and patellofemoral tracking were carefully assessed before wound closure.

Perioperative management followed a standardized institutional protocol. All patients received prophylactic antibiotics administered within one hour before skin incision and continued according to institutional guidelines. Venous thromboembolism prophylaxis was prescribed postoperatively in accordance with current recommendations.

Postoperative rehabilitation was standardized for all participants. Early mobilization was initiated on the first postoperative day under the supervision of a physiotherapist. Weight-bearing as tolerated was encouraged immediately after surgery. During hospitalization, patients participated in supervised physiotherapy twice daily, focusing on pain control, edema reduction, restoration of knee range of motion, quadriceps activation, gait re-education with progressive assistive device weaning, transfer training, and functional mobility exercises. After discharge, patients completed a structured outpatient rehabilitation program consisting of approximately three supervised sessions per week for 8 weeks.

Rehabilitation included progressive range-of-motion exercises, quadriceps and hamstring strengthening, balance and proprioceptive training, stair-climbing practice, gait training, and functional exercises aimed at restoring activities of daily living. Patients were additionally instructed to perform a standardized home exercise program daily, consisting of active and passive knee range-of-motion exercises, straight-leg raises, quadriceps isometric contractions, heel slides, ankle pumps, and progressive walking exercises. Rehabilitation progression was individualized according to patient tolerance while following the same institutional rehabilitation protocol.

Attendance at supervised outpatient physiotherapy sessions was monitored using institutional attendance records. The mean attendance rate was 91%, with 132 of 150 participants (88.0%) attending at least 80% of the prescribed sessions. Compliance with the prescribed home exercise program was assessed by patient self-report during follow-up visits, with 125 participants (83.3%) reporting regular adherence throughout the rehabilitation period. Rehabilitation was discontinued prematurely in 4 participants (2.7%), and minor deviations from the standardized rehabilitation protocol were documented in 7 participants (4.7%), primarily because of temporary medical conditions or personal scheduling constraints.

The same standardized postoperative rehabilitation protocol was applied to all participants throughout the study period to minimize treatment-related variability and ensure consistency in postoperative management, thereby facilitating longitudinal assessment of gait recovery.

### 2.3. Gait Analysis

Spatiotemporal gait parameters were assessed using a Footscan^®^ pressure platform (RSscan International, Olen, Belgium) integrated within a computerized gait analysis system. The platform measured approximately 0.5 m in length (488 × 325 mm), contained 4096 pressure sensors (sensor density: 2 sensors/cm^2^), and acquired plantar pressure data at a sampling frequency of 250 Hz. Before each assessment session, the system was calibrated according to the manufacturer’s instructions to ensure accurate pressure measurements and consistent data acquisition.

Previous validation studies have demonstrated that pressure-based gait analysis systems provide good-to-excellent test–retest reliability for spatiotemporal gait and plantar pressure variables under standardized testing conditions, with intraclass correlation coefficients (ICCs) generally ranging from 0.75 to 0.90 for walking speed, step length, cadence, and plantar pressure measurements. Published validation studies of the Footscan^®^ system have reported low measurement error together with excellent intra-rater and inter-rater reliability for spatiotemporal gait variables, with intraclass correlation coefficients generally exceeding 0.85 under standardized testing conditions [49,50]. Evaluations were performed preoperatively and at 3, 6, and 12 months following TKA.

All assessments were conducted under standardized laboratory conditions by trained examiners. Patients were instructed to walk at a self-selected comfortable speed along a level walkway while wearing their usual footwear. Prior to data collection, participants were allowed several familiarization trials to minimize the influence of learning effects.

For each assessment session, three valid walking trials were recorded, and the mean value was used for statistical analysis. The following spatiotemporal gait parameters were analyzed:

- Walking speed (m/s);
- Step length (cm);
- Cadence (steps/min);
- Stance phase symmetry (%);
- SI (%);
- Single-limb support time (%);
- Interlimb loading asymmetry (%).

A trial was considered valid when the participant walked at a self-selected speed without targeting the platform and complete foot contact of each limb was recorded during the stance phase.

Walking speed was defined as the average forward velocity achieved during gait. Step length was measured as the linear distance between successive foot contacts of opposite limbs, while cadence was calculated as the number of steps performed per minute.

The Footscan^®^ pressure platform was used to record plantar pressure distribution and dynamic weight-bearing characteristics during walking. Bilateral loading data obtained during the stance phase were subsequently analyzed to calculate symmetry-related parameters, including the SI and interlimb loading asymmetry. Symmetry measures were calculated by comparing the operated limb with the clinically evaluated contralateral limb. Greater SI values indicated a more symmetrical gait pattern, whereas smaller loading asymmetry values reflected a more balanced distribution of body weight between the operated and contralateral limbs.

To ensure measurement reliability, all assessments were performed using the same equipment, examiner training procedures, and standardized testing protocol throughout the study period. Because of the prospective longitudinal study design, the assessors were aware of the assessment time point but were not required to review participants’ previous gait analysis results during data collection. Furthermore, gait parameters were recorded automatically by the Footscan^®^ system using standardized acquisition and analysis procedures, thereby minimizing observer-dependent measurement bias.

### 2.4. Functional Assessment

Functional recovery was evaluated using a combination of performance-based and patient-reported outcome measures. Assessments were performed preoperatively and at 3, 6, and 12 months following TKA by trained investigators who were not involved in the surgical procedures.

1. Timed Up and Go (TUG) Test

Functional mobility was assessed using the TUG test, a validated and reliable measure of functional mobility in patients with KOA and following TKA. Previous studies have demonstrated excellent test–retest reliability for the TUG test, with intraclass correlation coefficients generally exceeding 0.90 under standardized testing conditions [51,52]. Participants were instructed to stand up from a standard chair, walk a distance of 3 m at a comfortable pace, turn around, return to the chair, and sit down. The time required to complete the task was recorded in seconds using a digital stopwatch. Three trials were performed, and the mean value was used for statistical analysis. Lower TUG values indicated better functional performance and mobility.

2. Western Ontario and McMaster Universities Osteoarthritis Index (WOMAC)

Patient-reported outcomes were assessed using the validated 24-item Likert version of the WOMAC, which evaluates pain (5 items), stiffness (2 items), and physical function (17 items) in patients with knee osteoarthritis and following total knee arthroplasty. Previous validation studies have demonstrated excellent test–retest reliability and responsiveness of the WOMAC for longitudinal assessment in patients with KOA and after TKA [53,54]. Each item was scored on a five-point Likert scale (0 = none to 4 = extreme), and the total WOMAC score was calculated according to the validated scoring system, with higher scores indicating greater pain, stiffness, and functional impairment.

3. Knee Society Score (KSS)

Knee-specific functional outcomes were assessed using the functional component of the KSS, a validated and reliable instrument for evaluating walking ability, stair climbing, and functional performance following TKA. Previous studies have also demonstrated good-to-excellent reliability and responsiveness of the KSS in patients undergoing TKA [55,56]. Greater KSS values indicate better functional performance and improved postoperative recovery.

All functional assessments were performed at each follow-up visit using standardized testing procedures to ensure consistency throughout the study period.

### 2.5. Symmetry Analysis

Gait symmetry was evaluated to quantify the restoration of balanced lower-limb function following TKA. Symmetry-related parameters were derived from the spatiotemporal gait data and baropodometric measurements obtained during each assessment session.

The primary symmetry outcome was the SI, which reflects the degree of similarity between the operated and contralateral limbs during gait. Greater SI values indicate a more symmetrical gait pattern and improved biomechanical function. In contrast, smaller values suggest persistent gait asymmetry and altered weight-bearing behavior.

Interlimb loading asymmetry was assessed using plantar pressure distribution data recorded during walking. This parameter represents the percentage difference in load distribution between the operated and non-operated limbs during the stance phase of gait. Smaller loading asymmetry values indicate a more balanced weight-bearing pattern.

Single-limb support time was also analyzed as an indicator of confidence and stability during gait. This parameter reflects the percentage of the gait cycle during which the body is supported by a single limb. Improvements in single-limb support time are associated with enhanced functional stability and reduced compensatory gait strategies.

The SI was calculated according to the following formula:

SI = (1 − |XR − XL|/(0.5 × (XR + XL))) × 100(1)

XR and XL represent the measured values for the right and left lower limbs, respectively. A value of 100% represents perfect symmetry between limbs.

All symmetry-related parameters were evaluated preoperatively and at 3, 6, and 12 months postoperatively using the same assessment protocol and equipment to ensure measurement consistency throughout the study period.

### 2.6. Statistical Analysis

Statistical analyses were performed using IBM SPSS Statistics software (Version 29.0; IBM Corp., Armonk, NY, USA). Continuous variables were expressed as mean ± standard deviation (SD), whereas categorical variables were presented as frequencies and percentages.

The normality of data distribution was assessed using the Shapiro–Wilk test. The assumption of sphericity was evaluated using Mauchly’s test. When the assumption of sphericity was violated, Greenhouse–Geisser corrected degrees of freedom were applied. Changes in spatiotemporal gait parameters, symmetry-related variables, and functional outcomes across the four assessment time points (preoperative, 3 months, 6 months, and 12 months) were analyzed using repeated-measures analysis of variance (ANOVA). When significant differences were identified, Bonferroni-adjusted post hoc comparisons were performed to determine pairwise differences between assessment time points.

Effect sizes for repeated-measures ANOVA were calculated using partial eta squared (η^2^p) and interpreted according to conventional thresholds, with values of 0.01, 0.06, and 0.14 representing small, medium, and large effects, respectively.

Pearson correlation analysis was performed using the 12-month postoperative data to evaluate the relationships between gait biomechanics and clinical outcome measures at the final follow-up assessment. Correlations were calculated using one observation per participant; repeated measurements obtained at different follow-up time points were not pooled for correlation analyses. Correlation coefficients were interpreted according to standard criteria, with values of 0.10–0.29 considered weak, 0.30–0.49 moderate, and ≥0.50 strong correlations.

To identify independent predictors of postoperative gait recovery, a multivariate linear regression analysis was performed using walking speed at 12 months as the dependent variable. Predictor variables included preoperative walking speed, age, BMI, and baseline WOMAC score. Predictor variables were selected a priori based on their established clinical relevance and previous evidence demonstrating associations with postoperative functional recovery following TKA. To reduce the risk of model overfitting, only a limited number of clinically relevant predictors were included in the exploratory multivariable model. Specifically, preoperative walking speed, age, BMI, and baseline WOMAC score were selected because they have consistently been associated with postoperative functional recovery in previous studies. Accordingly, the regression analysis should be interpreted as exploratory rather than as a comprehensive predictive model. Multicollinearity was assessed using variance inflation factor (VIF) values, with VIF values < 5 considered acceptable.

Missing outcome data resulting from loss to follow-up were handled using a complete-case analysis. Only participants with complete data for the corresponding assessment time point were included in the longitudinal and multivariate analyses. Consequently, the final regression model was based on the 142 patients who completed the 12-month follow-up evaluation. No imputation of missing data was performed because the proportion of missing observations was low (5.3%), and the reasons for loss to follow-up were documented. To evaluate potential attrition bias, baseline demographic and clinical characteristics of participants who completed the study were compared with those of participants lost to follow-up using appropriate statistical tests. No statistically significant baseline differences were identified (all *p* > 0.05), supporting the assumption that missingness was unlikely to introduce substantial attrition bias.

The primary analyses evaluated longitudinal changes in spatiotemporal gait parameters and symmetry-related outcomes following TKA using repeated-measures ANOVA. Secondary analyses assessed changes in functional outcome measures (TUG, WOMAC, and KSS) and the associations between biomechanical and clinical variables using Pearson correlation analysis. Multivariate linear regression was performed as an exploratory analysis to identify independent predictors of walking speed at 12 months after surgery.

A priori sample size estimation was performed using G*Power software (version 3.1.9.7; Heinrich Heine University Düsseldorf, Düsseldorf, Germany). Because the primary analysis evaluated longitudinal changes in walking speed across four assessment time points (preoperative and 3, 6, and 12 months postoperatively), the calculation was based on the F tests–ANOVA: Repeated measures, within factors procedure. In the absence of a directly comparable published estimate for this study design, a conventional medium effect size (Cohen’s f = 0.25) was assumed. The analysis further assumed a significance level (α) of 0.05, statistical power of 80%, one study group, four repeated measurements, a correlation of 0.50 among repeated measurements, and a nonsphericity correction (ε) of 0.75. Under these assumptions, the minimum required sample size was estimated to be approximately 22 participants. A total of 150 patients were enrolled, and complete 12-month follow-up data were available for 142 participants, substantially exceeding the estimated minimum sample size.

Statistical significance was established at a two-sided *p*-value < 0.05. Where appropriate, results, including those from the multivariable regression analysis, are presented with 95% confidence intervals (95% CIs) to provide an estimate of the precision of the observed effects.

### 2.7. Ethical Considerations

The study protocol was reviewed and approved by the Ethics Committee of Arad County Emergency Clinical Hospital, Arad, Romania (Approval No. 81/1/1/6 June 2024). The study was conducted in accordance with the ethical principles of the Declaration of Helsinki and complied with the provisions of the General Data Protection Regulation. This investigator-initiated prospective observational cohort study was not prospectively registered in a public study registry.

Written informed consent was obtained from all participants prior to enrollment. All collected data were anonymized before analysis to ensure participant confidentiality and data protection throughout the study.

## 3. Results

### 3.1. Patient Characteristics

A total of 150 patients undergoing primary TKA for end-stage KOA were included in the study. Follow-up data at 12 months were available for 142 patients (94.7%).

Eight participants were lost to follow-up during the study period. Three participants were lost before the 6-month assessment because of withdrawal of consent (*n* = 2) and medical complications (*n* = 1). An additional five participants were lost before the 12-month assessment because of inability to attend follow-up visits (*n* = 2), medical complications (*n* = 1), relocation (*n* = 1), and withdrawal of consent (*n* = 1). Comparison of baseline demographic and clinical characteristics between participants who completed the study and those lost to follow-up showed no statistically significant differences (Table 1).

The cohort consisted of 96 women (64.0%) and 54 men (36.0%), with a mean age of 67.8 ± 6.9 years and a mean BMI of 30.7 ± 4.2 kg/m^2^. Baseline demographic and clinical characteristics of the study population are summarized in Table 2.

### 3.2. Changes in Spatiotemporal Gait Parameters

Significant improvements were observed in all spatiotemporal gait parameters throughout the follow-up period. Walking speed increased from 0.84 ± 0.16 m/s preoperatively to 1.21 ± 0.18 m/s at 12 months postoperatively (*p* < 0.001). Similarly, step length increased from 49.8 ± 7.4 cm to 64.0 ± 6.4 cm, while cadence improved from 91 ± 10 to 108 ± 8 steps/min. Repeated-measures ANOVA confirmed significant changes over time for all investigated gait parameters (all *p* < 0.001). Detailed changes in gait parameters during follow-up, including effect size estimates, are presented in Table 3.

**Figure 2:**
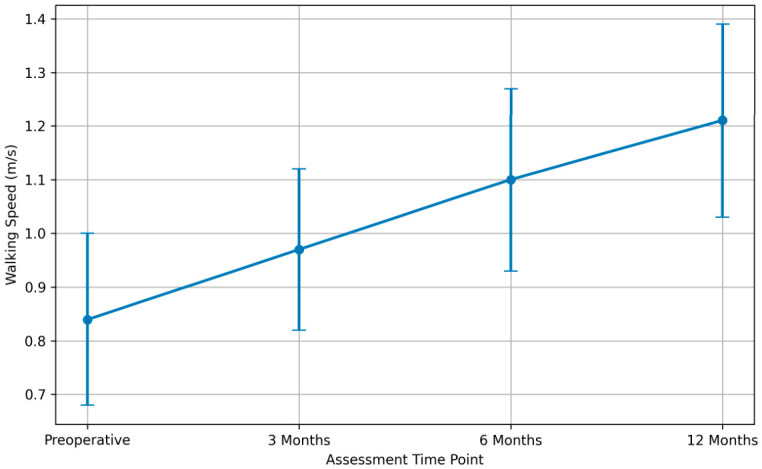
Changes in walking speed following TKA during the first postoperative year. Data are presented as mean ± SD. Error bars represent SD. Sample sizes were as follows: preoperative (*n* = 150), 3 months (*n* = 150), 6 months (*n* = 147), and 12 months (*n* = 142).

Mean walking speed increased progressively from 0.84 m/s preoperatively to 0.97 m/s at 3 months, 1.10 m/s at 6 months, and 1.21 m/s at 12 months postoperatively, demonstrating a significant improvement in gait performance during the first postoperative year (Figure 2).

**Figure 3:**
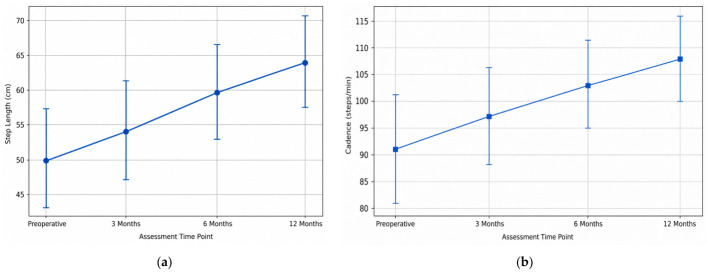
Changes in spatiotemporal gait parameters following TKA during the first postoperative year. (**a**) Mean step length and (**b**) mean cadence at each assessment time point (preoperative, 3 months, 6 months, and 12 months). Data are presented as mean ± SD. Error bars represent SD. Sample sizes were: preoperative (*n* = 150), 3 months (*n* = 150), 6 months (*n* = 147), and 12 months (*n* = 142).

Step length and cadence also demonstrated progressive improvements throughout follow-up, reflecting enhanced gait efficiency and functional recovery after surgery. The temporal evolution of these parameters is presented in Figure 3.

### 3.3. Symmetry Restoration Following TKA

Symmetry-related gait parameters improved progressively throughout the postoperative follow-up period (Table 4). The mean symmetry index (SI) increased from 73 ± 11% preoperatively to 81 ± 10% at 3 months, 88 ± 8% at 6 months, and 93 ± 6% at 12 months (*p* < 0.001), indicating gradual restoration of gait symmetry following TKA.

Similarly, interlimb loading asymmetry decreased significantly over time, from 18.2 ± 6.1% before surgery to 12.9 ± 5.0% at 3 months, 7.6 ± 3.4% at 6 months, and 4.8 ± 2.3% at the final follow-up (*p* < 0.001), reflecting progressively more balanced weight-bearing between the operated and contralateral limbs.

Single-limb support time also demonstrated significant improvement, increasing from 34.1 ± 4.8% preoperatively to 36.5 ± 4.3%, 38.2 ± 3.9%, and 39.9 ± 3.6% at 3, 6, and 12 months, respectively (*p* < 0.001). Collectively, these findings indicate progressive restoration of lower-limb symmetry and weight-bearing capacity during the first postoperative year following TKA.

**Figure 4:**
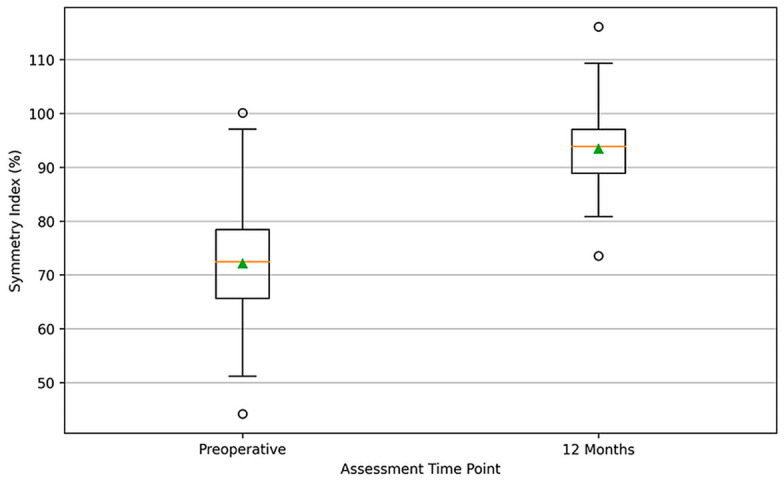
Distribution of SI values before TKA and at the 12-month postoperative assessment. The box represents the interquartile range (IQR), the horizontal line indicates the median, the green triangle indicates the mean, whiskers extend to 1.5 × IQR, and circles represent outliers. Sample sizes were: preoperative (*n* = 150) and 12 months (*n* = 142).

The distribution of SI values before surgery and at 12 months postoperatively is illustrated in Figure 4. The figure demonstrates a clear shift toward greater symmetry following TKA, with both the median and mean SI values increasing substantially at 12 months compared with the preoperative assessment. In addition, the postoperative distribution is characterized by a narrower interquartile range, indicating reduced variability in gait symmetry among participants, although a small number of outliers remained.

### 3.4. Functional Outcomes

Marked functional improvements were observed during follow-up. The TUG test improved from 15.9 ± 3.4 s preoperatively to 8.4 ± 1.8 s at 12 months (*p* < 0.001). Similarly, WOMAC scores improved by approximately 72%, while KSS functional scores increased significantly throughout the study period. Detailed functional outcomes are presented in Table 5.

**Figure 5:**
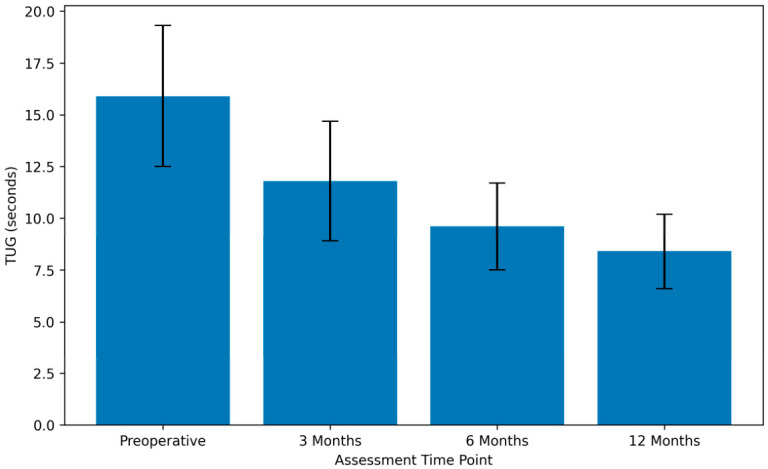
Changes in TUG performance following TKA during the first postoperative year. Data are presented as mean ± SD. Error bars represent SD. Sample sizes were: preoperative (*n* = 150), 3 months (*n* = 150), 6 months (*n* = 147), and 12 months (*n* = 142).

The temporal improvement in TUG performance during follow-up is illustrated in Figure 5. A progressive reduction in TUG completion time was observed across all follow-up assessments, indicating continuous improvement in functional mobility after TKA. The greatest improvement occurred during the early postoperative period, with further gains observed between 3 and 12 months, reflecting sustained recovery throughout the first postoperative year.

### 3.5. Correlation Analysis

Significant correlations were identified between biomechanical and clinical outcomes. Walking speed showed a strong negative correlation with WOMAC score (r = −0.74, *p* < 0.001), indicating that patients with smaller pain and disability exhibited superior gait performance. Similarly, walking speed was strongly correlated with TUG performance (r = −0.79, *p* < 0.001), while moderate negative correlations were observed between walking speed and both BMI (r = −0.41, *p* < 0.001) and age (r = −0.38, *p* < 0.001). Detailed correlation coefficients are presented in Table 6.

**Figure 6:**
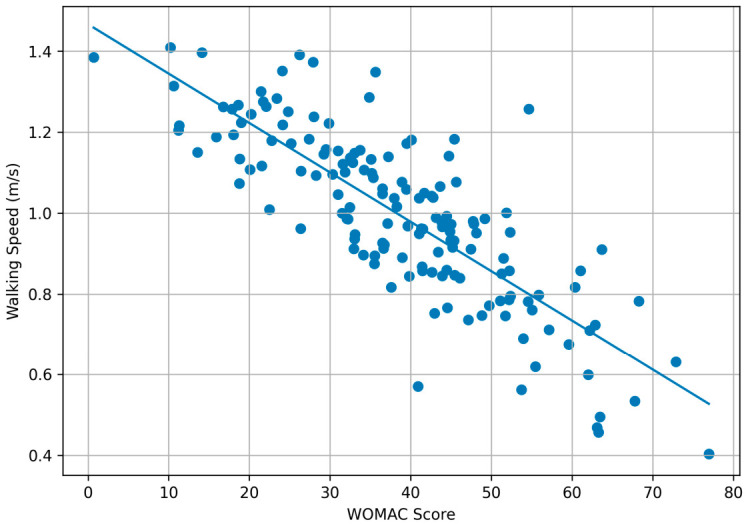
Scatter plot illustrating the relationship between walking speed and WOMAC score at the 12-month postoperative assessment. Each point represents one participant. The solid line represents the linear regression trend derived from the Pearson correlation analysis. The analysis included 142 participants with complete 12-month follow-up data (*n* = 142).

The relationship between walking speed and WOMAC score is illustrated in Figure 6. The scatter plot demonstrates a clear inverse linear association, with greater WOMAC scores being associated with reduced walking speeds. Although some variability is evident among individual participants, the regression line indicates a consistent trend whereby poorer patient-reported outcomes correspond to reduced gait performance.

### 3.6. Predictors of Gait Recovery

A multivariate linear regression model was performed to identify independent predictors of walking speed at 12 months following TKA. The model explained 48% of the variance in postoperative walking speed (Adjusted R^2^ = 0.48). Preoperative walking speed emerged as the strongest predictor of gait recovery (β = 0.47, *p* < 0.001), while BMI and age were identified as significant negative predictors. No evidence of multicollinearity was observed among the independent variables, with all VIF values below the commonly accepted threshold of 2.0. Detailed results of the regression analysis are presented in Table 7.

**Figure 7:**
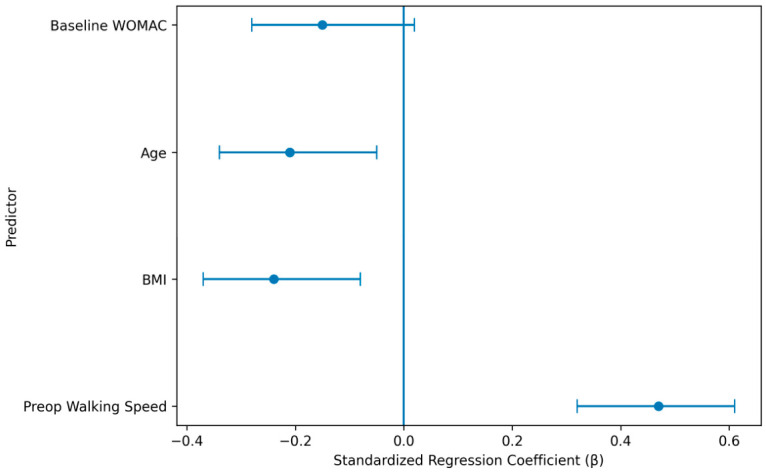
Forest plot showing standardized regression coefficients (β) with 95% confidence intervals from the multivariate linear regression model predicting walking speed at the 12-month postoperative assessment. Positive coefficients indicate a positive association with postoperative walking speed, whereas negative coefficients indicate an inverse association. The analysis included 142 participants with complete 12-month follow-up data (*n* = 142).

The magnitude and direction of the associations between the investigated predictors and gait recovery are illustrated in Figure 7. The forest plot demonstrates that preoperative walking speed showed the strongest positive association with postoperative gait recovery, whereas age, BMI, and baseline WOMAC score were negatively associated with walking speed. The standardized regression coefficients and their corresponding 95% confidence intervals illustrate both the direction and relative magnitude of these associations.

## 4. Discussion

Despite the well-established benefits of total TKA for pain relief and functional improvement, the extent to which normal gait biomechanics and symmetry are restored after surgery remains incompletely understood. Previous studies have reported variable recovery trajectories and have often focused on isolated gait parameters or patient-reported outcomes, with relatively limited evidence integrating objective gait biomechanics, symmetry restoration, functional recovery, and predictive factors within a prospective longitudinal design. A better understanding of these relationships is clinically important because objective gait assessment may facilitate identification of patients at risk of delayed recovery, support individualized rehabilitation strategies, and ultimately improve long-term functional outcomes.

The present prospective longitudinal cohort study was therefore undertaken to evaluate changes in gait biomechanics, symmetry restoration, and functional recovery during the first postoperative year following primary TKA. The principal findings were fourfold. First, significant improvements were observed in all spatiotemporal gait parameters, including walking speed, step length, cadence, and stance phase symmetry. Second, substantial restoration of gait symmetry occurred during follow-up, accompanied by marked reductions in interlimb loading asymmetry. Third, biomechanical improvements were strongly associated with clinical outcomes. Finally, preoperative walking speed emerged as the strongest independent predictor of postoperative gait recovery. Collectively, these findings support all four study hypotheses and further emphasize the value of objective gait assessment in evaluating recovery following TKA.

### 4.1. Interpretation of Changes in Spatiotemporal Gait Parameters

One of the most important findings of the present study was the progressive improvement in spatiotemporal gait parameters throughout the first postoperative year. Walking speed increased from 0.84 m/s preoperatively to 1.21 m/s at 12 months, accompanied by significant increases in step length and cadence. These results are consistent with previous investigations demonstrating that patients undergoing TKA experience substantial improvements in locomotor performance and gait efficiency [57,58,59,60].

Walking speed is widely regarded as a robust indicator of functional mobility and overall health status. Previous studies have reported that patients with advanced KOA frequently exhibit slower gait velocity due to pain, joint instability, and altered movement strategies [61,62,63,64]. Following arthroplasty, patients generally experience pain reduction and improved joint mechanics, which are associated with more efficient ambulation, allowing patients to gradually recover more physiological gait patterns. The magnitude of improvement observed in the present study is comparable to findings reported in contemporary gait analysis studies following TKA [58,60,65].

Interestingly, improvements were progressive across all follow-up intervals rather than occurring immediately after surgery. This observation suggests that gait recovery continues beyond the early postoperative period and likely reflects ongoing neuromuscular adaptation, muscle strengthening, and rehabilitation-related improvements in movement control.

### 4.2. Interpretation of Symmetry Restoration Following TKA

Restoration of gait symmetry represents another important finding of the present investigation. SI values increased substantially during follow-up, whereas interlimb loading asymmetry decreased markedly. These findings suggest progressive normalization of weight-bearing behavior and biomechanical function following surgery.

Persistent gait asymmetry is a well-recognized consequence of advanced KOA and may remain present even after successful arthroplasty [28,32,34,45,59]. Several authors have suggested that asymmetric loading patterns contribute to inefficient movement, increased metabolic cost of walking, and excessive mechanical stress on the contralateral limb [28,57,58,59,60,61,62,63,64,65,66,67,68]. Therefore, restoration of symmetry may represent a clinically meaningful indicator of functional recovery beyond traditional patient-reported outcomes.

The observed improvements are consistent with previous reports demonstrating enhanced bilateral weight distribution following TKA [44,69,70,71]. The concomitant increase in single-limb support time further supports the hypothesis that patients progressively regained confidence in the operated limb and adopted more balanced gait patterns during the recovery process.

### 4.3. Functional Recovery and Clinical Outcomes

Substantial improvements were also observed in functional outcome measures. TUG performance improved significantly throughout follow-up, while WOMAC scores decreased and KSS functional scores increased. Collectively, these findings indicate marked improvements in mobility, pain, and overall functional capacity.

The magnitude of WOMAC improvement observed in the present study is comparable to previously reported outcomes following TKA [72,73,74,75]. Similarly, the reduction in TUG completion time reflects enhanced dynamic balance, lower-limb strength, and walking ability. Because the TUG test evaluates multiple components of functional mobility, including standing, walking, turning, and sitting, it provides clinically relevant information regarding recovery of everyday activities [52,76,77,78,79,80].

The improvements observed in the present study also appear to be clinically meaningful when interpreted relative to previously published minimal clinically important difference (MCID) values. Previous studies have established MCID thresholds for the WOMAC and KSS, as well as minimal detectable change values for the TUG test following TKA [81,82]. In the present cohort, the marked reduction in WOMAC scores, increase in KSS functional scores, and decrease in TUG completion time exceeded these published thresholds, indicating that the observed improvements were not only statistically significant but also clinically meaningful from the patient’s perspective.

The parallel improvements observed in objective gait parameters and patient-reported outcomes support the concept that biomechanical recovery and functional recovery are closely interconnected following arthroplasty.

### 4.4. Interpretation of Predictors of Gait Recovery

An important contribution of the present study is the identification of predictors associated with postoperative gait recovery. Multivariate regression analysis demonstrated that preoperative walking speed was the strongest independent predictor of walking performance at 12 months. In contrast, increased age and greater BMI were associated with less favorable outcomes.

These findings are consistent with previous studies indicating that baseline functional status is a major determinant of postoperative recovery [58,83,84,85,86]. Patients with better preoperative mobility may possess greater muscle strength, better neuromuscular control, and higher physiological reserve, facilitating more efficient postoperative rehabilitation. Conversely, obesity and advanced age have been associated with slower recovery trajectories and reduced functional gains following TKA [58,87,88,89,90].

The identification of these predictors has important clinical implications because it may facilitate risk stratification and individualized rehabilitation planning. Patients presenting with poorer preoperative gait performance may benefit from targeted prehabilitation programs designed to optimize postoperative recovery.

### 4.5. Clinical Implications

The present findings highlight the value of objective gait assessment as a complementary tool to conventional clinical outcome measures following TKA. Although patient-reported outcome measures such as WOMAC and the KSS provide important information regarding pain, symptoms, and perceived function, they may not fully capture residual biomechanical impairments that persist despite successful surgery. In contrast, quantitative gait analysis enables objective evaluation of walking performance, movement quality, weight-bearing symmetry, and locomotor efficiency, thereby providing a more comprehensive assessment of postoperative recovery.

The significant improvements observed in walking speed, step length, cadence, and symmetry-related parameters suggest that objective gait metrics may serve as sensitive markers of observed functional recovery throughout the rehabilitation process. Furthermore, the strong correlations identified between gait parameters and clinical outcomes indicate that biomechanical improvements are closely associated with reductions in pain and disability. These findings support the integration of gait analysis into routine postoperative monitoring, particularly in patients demonstrating delayed functional recovery or persistent mobility limitations.

The identification of preoperative walking speed as the strongest predictor of postoperative gait performance also has important practical implications. Patients presenting with reduced mobility before surgery may represent a subgroup at increased risk of suboptimal recovery and may therefore benefit from targeted prehabilitation programs aimed at improving strength, balance, and walking capacity before arthroplasty. Early recognition of these patients could facilitate individualized treatment strategies and optimize postoperative outcomes.

In addition, the marked restoration of gait symmetry observed during follow-up emphasizes the importance of rehabilitation programs specifically designed to address asymmetrical loading patterns and compensatory movement strategies. Persistent gait asymmetry has been associated with increased mechanical stress on the contralateral limb, impaired functional performance, and reduced movement efficiency. Consequently, interventions focusing on weight-bearing symmetry, neuromuscular control, and gait re-education may contribute to more complete biomechanical recovery after TKA. Although the observational design of the present study precludes definitive conclusions regarding the factors responsible for the favorable recovery observed, several aspects of the standardized postoperative management may have contributed to these outcomes. All participants followed the same structured rehabilitation protocol, which combined supervised physiotherapy with a daily home exercise program emphasizing progressive range-of-motion exercises, muscle strengthening, gait re-education, balance training, and functional mobility. The combination of supervised rehabilitation, continued home-based exercise, and progressive weight-bearing may have facilitated the gradual improvements in gait biomechanics and functional performance observed throughout the first postoperative year. Nevertheless, future prospective studies are warranted to determine the relative contribution of these individual components to postoperative recovery.

From a broader healthcare perspective, objective gait assessment may facilitate more personalized rehabilitation pathways and support evidence-based decision-making. The incorporation of biomechanical evaluation into clinical practice may improve patient stratification, enable earlier identification of individuals requiring intensified rehabilitation, and ultimately contribute to enhanced long-term functional outcomes and patient satisfaction following TKA.

### 4.6. Strengths and Limitations

Several strengths of the present study should be acknowledged. First, the prospective longitudinal design allowed detailed evaluation of postoperative recovery trajectories over multiple assessment time points during the first year following TKA. This design enabled the identification of temporal changes in gait biomechanics, symmetry restoration, and functional outcomes, providing a more comprehensive understanding of recovery patterns than cross-sectional analyses.

Second, the study integrated objective biomechanical assessment with validated clinical outcome measures, including the TUG test, WOMAC, and KSS, each of which has demonstrated validity and reliability in patients with KOA and following TKA. The combination of spatiotemporal gait analysis, symmetry-related parameters, functional performance testing, and patient-reported outcome measures allowed a multidimensional evaluation of postoperative recovery. Such an approach provides complementary information regarding both biomechanical function and patient-perceived outcomes, thereby enhancing the clinical relevance of the findings.

Third, the study achieved an excellent follow-up rate (94.7%), minimizing the risk of attrition bias and strengthening the reliability of the longitudinal analyses. In addition, all participants underwent a standardized surgical and rehabilitation protocol, reducing treatment-related variability and facilitating more accurate assessment of recovery-related changes. Furthermore, the inclusion of correlation and multivariate regression analyses enabled the identification of clinically relevant factors associated with postoperative gait recovery. Although the proportion of missing data was low (5.3%), attrition bias cannot be completely excluded if participants lost to follow-up differed systematically from those who completed the study.

Despite these strengths, several limitations should be considered when interpreting the findings. First, the study was conducted at a single tertiary orthopedic center, which may limit the generalizability of the results to other institutions, healthcare systems, or patient populations. Multicenter studies involving more diverse cohorts are needed to confirm the external validity of the present findings.

Second, although the sample size was sufficient to detect significant differences across the investigated outcomes, larger cohorts may allow more detailed subgroup analyses according to age, sex, BMI, implant characteristics, or rehabilitation strategies. Such analyses may further improve understanding of factors influencing postoperative recovery.

Third, follow-up was limited to 12 months after surgery. While substantial improvements were observed during this period, longer-term follow-up may be necessary to determine whether biomechanical recovery continues beyond the first postoperative year and whether restored gait patterns are maintained over time.

Fourth, gait assessment was primarily based on spatiotemporal and symmetry-related parameters. Although these measures provide clinically meaningful information, additional kinetic and three-dimensional kinematic analyses could offer a more comprehensive evaluation of lower-limb biomechanics.

In addition, patellar resurfacing was performed selectively according to predefined intraoperative clinical criteria rather than as a uniform procedure for all patients. Although this approach reflects routine clinical practice, it may have introduced some treatment heterogeneity that could have influenced postoperative biomechanical and functional outcomes.

Furthermore, several potentially relevant demographic, clinical, perioperative, and rehabilitation-related variables, including sex, anesthesia type, selective patellar resurfacing, rehabilitation adherence, pain medication use, prehabilitation exposure, and the severity of degenerative changes in the contralateral knee, were not included as covariates in the multivariate regression model. Consequently, residual confounding cannot be excluded and should be considered when interpreting the findings. Although patients with symptomatic contralateral KOA requiring surgical treatment were excluded, mild to moderate degenerative changes in the non-operated knee may have influenced symmetry-related outcomes. In addition, the absence of an age-matched healthy control group precluded direct comparison of postoperative gait biomechanics and symmetry indices with normative values, thereby limiting assessment of the extent to which gait patterns normalized following TKA.

Finally, the observational design of the study precludes definitive conclusions regarding causal relationships between biomechanical and clinical variables. Although significant associations were identified, further interventional studies are required to determine whether targeted rehabilitation strategies aimed at improving gait biomechanics can directly influence long-term functional outcomes following TKA.

### 4.7. Future Directions

Future research should investigate the long-term evolution of gait biomechanics following TKA and determine whether the improvements observed during the first postoperative year are maintained over time. Although significant recovery was demonstrated in the present study, the extent to which gait patterns normalize beyond 12 months remains incompletely understood. Longitudinal studies with extended follow-up periods may provide valuable information regarding the durability of biomechanical and functional improvements and their relationship with implant longevity and patient satisfaction.

Further investigations should also explore the role of advanced biomechanical assessment techniques in monitoring postoperative recovery. The integration of three-dimensional motion analysis, force-platform measurements, surface electromyography, and wearable sensor technologies may provide a more comprehensive characterization of movement patterns and neuromuscular adaptations following TKA. These approaches could facilitate the identification of subtle biomechanical deficits that are not detected through conventional clinical assessments and improve objective monitoring of functional recovery in both laboratory and real-world environments.

The increasing availability of digital health technologies also presents important opportunities for future research. Continuous gait monitoring using wearable devices, combined with machine-learning and artificial intelligence algorithms, may improve prediction of postoperative recovery trajectories and support the development of personalized rehabilitation programs tailored to individual patient characteristics.

Future studies should also investigate the effectiveness of targeted rehabilitation interventions designed to optimize gait recovery and symmetry restoration. Randomized controlled trials comparing conventional rehabilitation protocols with individualized gait-focused training programs may help determine whether specific therapeutic strategies can accelerate biomechanical recovery and improve long-term functional outcomes.

Another important area of investigation involves the identification and validation of predictive factors associated with successful recovery after TKA. Although preoperative walking speed emerged as a significant predictor in the present study, additional demographic, clinical, perioperative, rehabilitation-related, and biomechanical variables may also contribute to postoperative outcomes. Future studies should investigate the influence of factors including sex, anesthesia type, selective patellar resurfacing, rehabilitation adherence, pain medication use, prehabilitation exposure, and contralateral knee status on postoperative gait recovery and functional outcomes. Incorporating these variables into multivariable predictive models may improve patient stratification and facilitate more individualized rehabilitation strategies. In addition, future prospective studies should include age-matched healthy control participants to provide normative reference values for spatiotemporal gait parameters and symmetry indices, thereby enabling a more comprehensive evaluation of the extent to which postoperative gait patterns approach normal physiological function. Larger multicenter studies involving diverse patient populations are also warranted to externally validate predictive models and improve their generalizability.

Finally, future research should investigate the interactions between gait biomechanics, patient-reported outcomes, implant-related factors, and surgical techniques, including prosthesis design and alignment strategies. A better understanding of these relationships may facilitate optimization of both surgical management and postoperative rehabilitation, ultimately contributing to improved functional recovery and quality of life following TKA.

## 5. Conclusions

In this prospective longitudinal cohort study, patients undergoing primary TKA demonstrated significant improvements in gait biomechanics, symmetry restoration, and functional recovery during the first postoperative year. Progressive increases in walking speed, step length, cadence, and gait symmetry were observed throughout follow-up, accompanied by substantial improvements in functional outcomes, including TUG performance, WOMAC scores, and KSS. Furthermore, strong associations were identified between biomechanical gait parameters and clinical outcomes, highlighting the close relationship between objective gait recovery and patient function.

Preoperative walking speed emerged as the strongest independent predictor of postoperative gait performance, while increased age and greater BMI were associated with less favorable recovery trajectories. These findings suggest that objective gait assessment provides valuable information beyond conventional clinical outcome measures and may assist in identifying patients at risk of delayed recovery. Incorporating quantitative gait analysis into routine postoperative evaluation may facilitate individualized rehabilitation strategies and contribute to improved long-term functional outcomes following TKA.

## Figures and Tables

**Table 1:** Comparison of baseline characteristics between participants who completed the study and those lost to follow-up.

Variable	Completers (*n* = 142)	Non-Completers (*n* = 8)	*p*-Value
Age (years)	67.8 ± 6.8	68.1 ± 7.2	0.89
Female, *n* (%)	91 (64.1)	5 (62.5)	0.92
BMI (kg/m^2^)	30.6 ± 4.1	31.2 ± 4.5	0.67
Walking speed (m/s)	0.84 ± 0.16	0.82 ± 0.18	0.74
WOMAC score	67.2 ± 12.6	68.5 ± 13.4	0.81
TUG (s)	15.8 ± 3.3	16.1 ± 3.7	0.83

**Table 2:** Baseline demographic and clinical characteristics of the study population.

Variable	Value
Number of patients	150
Female, *n* (%)	96 (64.0)
Male, *n* (%)	54 (36.0)
Age (years)	67.8 ± 6.9
BMI (kg/m^2^)	30.7 ± 4.2
Kellgren-Lawrence Grade III	38 (25.3%)
Kellgren-Lawrence Grade IV	112 (74.7%)
Right TKA	81 (54.0%)
Left TKA	69 (46.0%)

**Table 3:** Evolution of gait parameters following TKA.

Parameter	Preoperative	3 Months	6 Months	12 Months	*p*-Value	Effect Size (η^2^p)
Walking speed (m/s)	0.84 ± 0.16	0.97 ± 0.15	1.10 ± 0.17	1.21 ± 0.18	<0.001	0.42
Step length (cm)	49.8 ± 7.4	54.3 ± 7.1	59.8 ± 6.7	64.0 ± 6.4	<0.001	0.37
Cadence (steps/min)	91 ± 10	97 ± 9	103 ± 8	108 ± 8	<0.001	0.31
Stance phase symmetry (%)	73 ± 11	81 ± 10	88 ± 8	93 ± 6	<0.001	0.29

Note: Data are presented as mean ± SD. Statistical comparisons were performed using repeated-measures ANOVA with Bonferroni-adjusted post hoc comparisons. Effect sizes are reported as partial eta squared (η^2^p).

**Table 4:** Symmetry-related gait parameters.

Parameter	Preoperative	3 Months	6 Months	12 Months	*p*-Value
SI (%)	73 ± 11	81 ± 10	88 ± 8	93 ± 6	<0.001
Loading asymmetry (%)	18.2 ± 6.1	12.9 ± 5.0	7.6 ± 3.4	4.8 ± 2.3	<0.001
Single-limb support (%)	34.1 ± 4.8	36.5 ± 4.3	38.2 ± 3.9	39.9 ± 3.6	<0.001

Note: Data are presented as mean ± SD. Differences across the four assessment time points (preoperative, 3 months, 6 months, and 12 months) were analyzed using repeated-measures ANOVA with Bonferroni-adjusted post hoc comparisons.

**Table 5:** Functional assessment outcomes.

Variable	Preoperative	3 Months	6 Months	12 Months	*p*-Value
TUG (seconds)	15.9 ± 3.4	11.8 ± 2.9	9.6 ± 2.1	8.4 ± 1.8	<0.001
WOMAC Score	67.3 ± 12.8	42.6 ± 11.7	28.5 ± 9.2	18.9 ± 7.4	<0.001
KSS Functional Score	44.8 ± 10.7	67.9 ± 9.8	79.1 ± 8.5	88.3 ± 7.2	<0.001

Note: Data are presented as mean ± SD. Statistical comparisons were performed using repeated-measures ANOVA with Bonferroni-adjusted post hoc comparisons.

**Table 6:** Pearson correlation coefficients.

Variables	r	*p*-Value
Walking Speed vs. WOMAC	−0.74	<0.001
Walking Speed vs. TUG	−0.79	<0.001
SI vs. WOMAC	−0.68	<0.001
BMI vs. Walking Speed	−0.41	<0.001
Age vs. Walking Speed	−0.38	<0.001

**Table 7:** Multivariate regression analysis.

Predictor	β	95% CI	*p*-Value	VIF
Preoperative Walking Speed	0.47	0.32–0.61	<0.001	1.42
BMI	−0.24	−0.37–−0.08	0.004	1.28
Age	−0.21	−0.34–−0.05	0.011	1.35
WOMAC Baseline	−0.15	−0.28–0.02	0.078	1.57

Note: β = standardized regression coefficient; CI = confidence interval; VIF = variance inflation factor. Adjusted R^2^ = 0.48. All VIF values were below 2.0, indicating no evidence of multicollinearity.

## Data Availability

The data supporting the findings of this study are available from the corresponding author upon reasonable request. The data are not publicly available due to privacy and ethical restrictions related to patient confidentiality.

## Abbreviations

The following abbreviations are used in this manuscript:

TKA	Total Knee Arthroplasty
TUG	Timed Up and Go test
WOMAC	Western Ontario and McMaster Universities Osteoarthritis Index
KSS	Knee Society Score
KOA	Knee Osteoarthritis
BMI	Body Mass Index
SI	Symmetry Index
ANOVA	Analysis of Variance
VIF	Variance Inflation Factor
SD	Standard Deviation
MCID	Minimal Clinically Important Difference
